# Multiple paravalvular leak 17 years after a second mitral valve replacement

**DOI:** 10.1186/1749-8090-8-24

**Published:** 2013-02-15

**Authors:** Tae-Eun Jung, Dong-Hyup Lee

**Affiliations:** 1 Department of Thoracic and Cardiovascular Surgery, College of Medicine, Yeungnam University, 317-1 Daemyung 5 Dong, Namgu, Daegu, Korea

**Keywords:** Heart, Valve, Surgery, Complications, Mitral valve replacement

## Abstract

Paravalvular leak (PVL) after prosthetic valve implantation is a significant complication and it usually occurs early in the postoperative period. We report a case of multiple PVL 17 years after the second mitral valve replacement without evidence of infection. The valve sutures were neither cut nor loosened. None of the sewing cuff of the mitral valve was covered with fibrous tissue. The sewing cuff was floated over the native annulus and large and multiple leakage was developed. The valve was easily removed and replaced with a new mechanical prosthesis.

## Background

Paravalvular leak (PVL) after prosthetic valve implantation is a significant complication and it usually occurs early in the postoperative period [1]. The patient underwent a mitral valve replacement two times before this operation, which makes this surgery a third-time operation. We report a rare case of multiple PVL 17 years after the second mitral valve replacement without evidence of infection.

## Case presentation

A 62-year-old female was admitted due to easy fatigue and dyspnea on exertion. She had undergone mitral valve replacement twice before this admission. The first operation was done with a tissue valve (29 mm, Carpentier-Edwards, Edwards Lifesciences, Irvine, CA, USA) 25 years before this admission. She suffered from degenerative changes in the valve 8 years after the first operation and underwent a redo mitral valve replacement with a mechanical prosthesis (29 mm, St. Jude Medical, Inc. St. Paul, MN, USA). She had been studied by serial transthoracic echocardiography (TTE) for years, but no sign of PVL had ever been detected. One year before the reoperation, when the mild dyspnea was first found, we were able to find the minimal multiple PVL by TTE. She had been treated with medication and her symptoms had improved.

After one year on medication, the patient was slightly icteric. A grade II/IV systolic murmur was heard at the apex. A chest radiograph showed the typical pattern of pulmonary edema and an increased cardiothoracic ratio of 65.0%. Her blood test revealed hemoglobin of 7.9 mg/dL and lactate dehydrogenase of 4,421 IU/L. Total bilirubin was 3.38 mg/dL and direct bilirubin was 0.58 mg/dL. The endoscopic examination did not reveal gastrointestinal bleeding or an ulcer, and the peripheral blood smear was compatible with hemolytic anemia.

**Figure 1:**
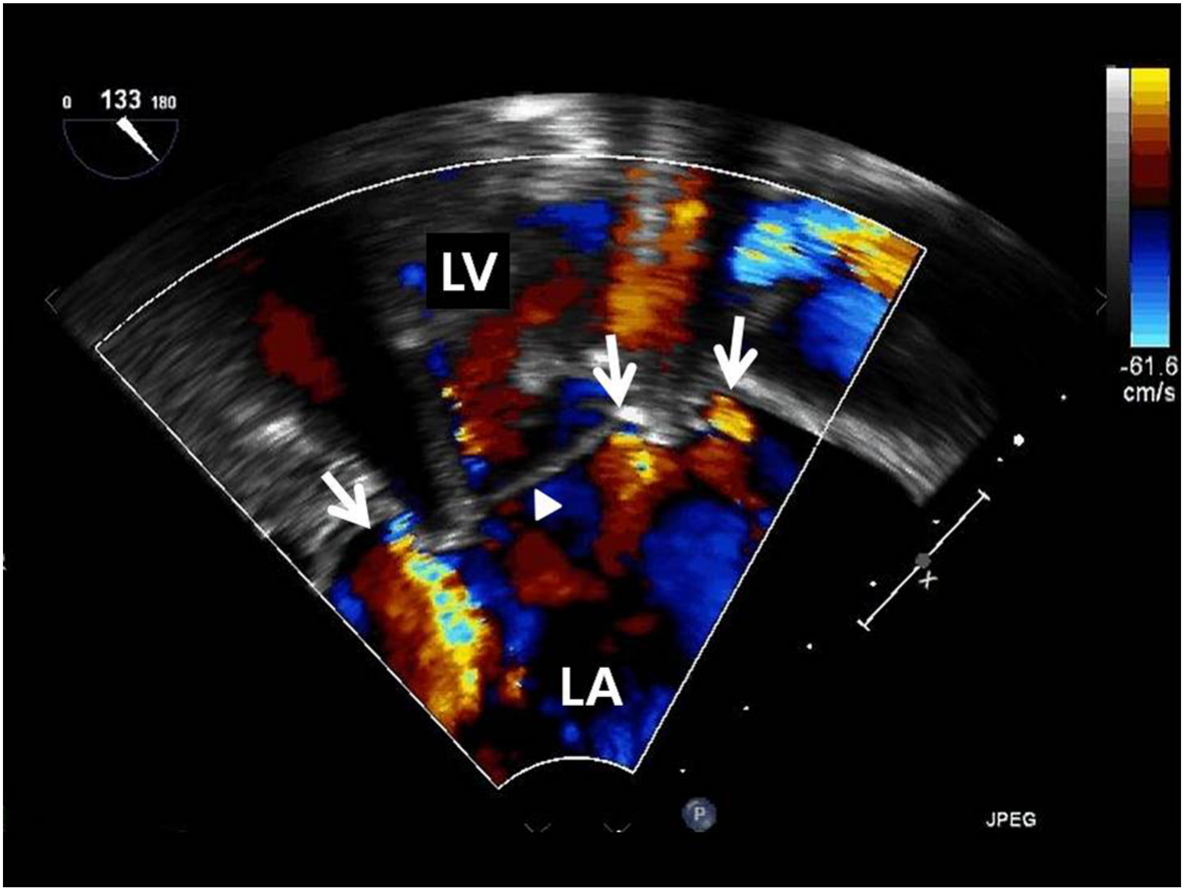
**Preoperative echocardiographic finding shows multiple paravalvular leaks in the mitral valve. **The arrow indicates multiple mitral regurgitant flow and the arrow head indicates the mechanical mitral valve (LA, left atrium; LV, left ventricle).

TTE revealed severe mitral regurgitation and moderate tricuspid regurgitation. The pulmonary artery pressure was 45 mmHg, and the left ventricular ejection fraction was 56%. Transesophageal echography (TEE) revealed PVL in multiple areas (Figure 1). Mitral regurgitation due to the PVL, causing heart failure and hemolytic anemia, was diagnosed, and we performed repeat open heart surgery for valve replacement.

**Figure 2:**
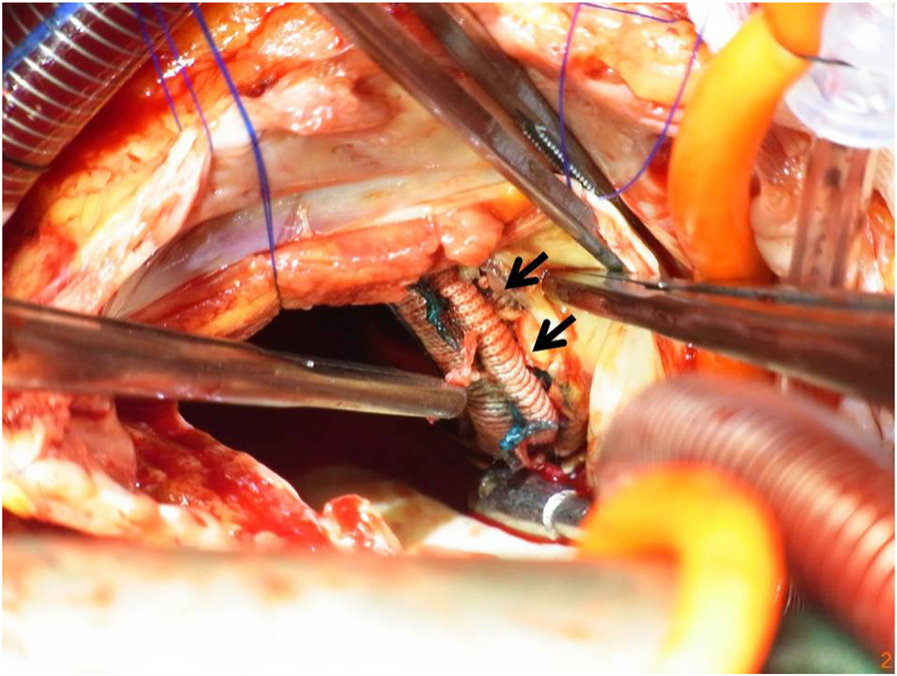
**Intraoperative finding shows the paravalvular leak area (arrow). **The sewing cuff was floating over the native annulus.

After a median sternotomy, the pericardium was carefully dissected. A standard cardiopulmonary bypass was established by bicaval cannulation and an aortic cannulation at the distal end of the ascending aorta. Cold blood cardioplegia was delivered with both the antegrade and subsequent retrograde methods. A right atriotomy and atrial septal incision was made for both mitral and tricuspid valve management. None of the sewing cuffs of the mitral valve were covered with fibrous tissue, and the neoendothelialization between the sewing cuff and annulus was not seen. There was no abscess or fragile tissue suggesting prosthetic valve endocarditis. The sutures were neither cut nor loosened. The sewing cuff was floated over the native annulus in multiple areas and lead to paravalvular leakage (Figure 2).

**Figure 3:**
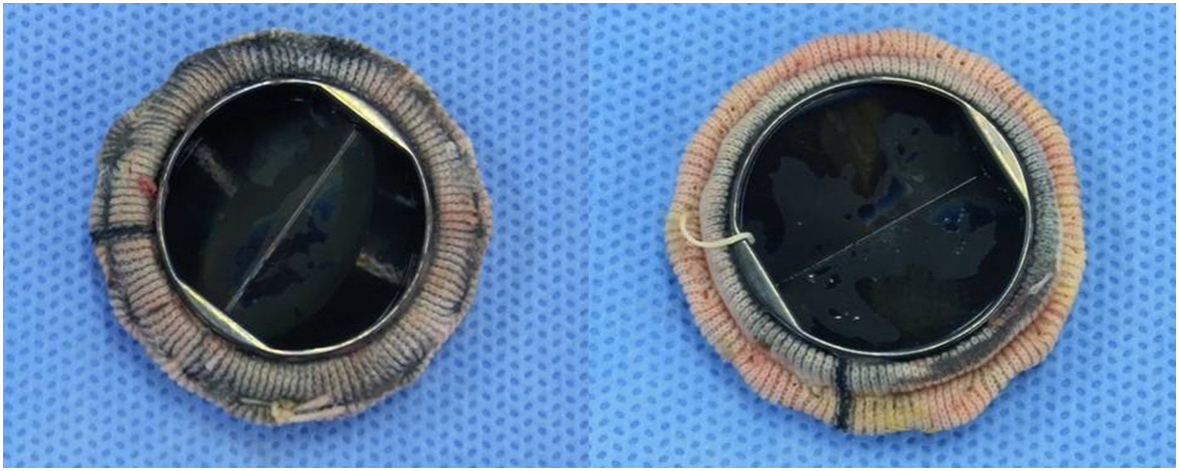
**Postoperative findings of removed valve. Not the entire sewing cuff of the mitral valve was covered with tissue, and the valve was removed easily. **(Left, left atrial side; Right, left ventricular side).

We planned to replace it with a new prosthesis instead of repairing it because the dehiscence sites were multiple and the remaining sutures looked weak. The valve was easily removed (Figure 3) and replaced with a new mechanical prosthesis (27 mm, ATS Medical, Inc.; Minneapolis, MN, USA) in the same annular position. The slightly deep everted mattress sutures were done while avoiding injury of important adjacent tissue. After closing the atrial septum, a tricuspid valve annuloplasty was performed with a ring (30 mm, MC3 ring, Edwards Lifesciences, Irvine, CA, USA). The cross-clamp time was 177 min, and the cardiopulmonary bypass time was 222 min.

The patient’s postoperative course was good, and she was extubated on the next day and transferred to the general ward two days after surgery. Postoperatively, the LDH decreased dramatically to 699 IU/l and the dyspnea was disappeared. TTE showed that the pressure of the right ventricle decreased to 30 mmHg, and there was no PVL. The patient’s postoperative recovery was uneventful, and she was discharged 9 days after surgery.

## Discussion

PVL is a rare complication after mitral valve replacement. The incidence of PVL is higher in mechanical valves than bioprosthesis [2]. The reoperation rate due to PVL after use of the Björk-Shiley prosthesis was reported at 2.2% [3] and at 2.5% in the isolated MVR of 372 patients [4]. Lindblom reported that the PVL in the mitral position was 3.5 times higher than in the aortic position in Björk-Shiley prostheses [5]. It occurs most frequently in the early postoperative period. Genoni et al. [1] reported a median time of 119 days (range: 1 day to 23 years) after primary mitral valve replacement in 96 patients.

Once PVL occurs, it may causes morbidity due to hemolytic anemia or heart failure. The most frequent cause of PVL was found to be fragile native tissue, such as is seen with endocarditis. Annular calcification and continuous running sutures in placing a prosthesis are associated with the development of PVL [6].

In our patient, there was no apparent annular calcification, and no stitch loosening or disruption. Furthermore, with the intraoperative findings and laboratory data of this case, an infection did not seem to be the cause. It was uncertain why PVL appeared a long time after second mitral valve replacement.

The only unusual finding was that not all of the entire sewing ring was covered by tissue, which was different than what is usually found in repeat operations and the sewing cuff was floated over the native annulus in multiple areas without cutting or loosening.

Minami et al. [7] proposed three possible causes of late PVL: (1) The suture site of the remnant valve tissue under the sewing cuff could have undergone long-term degenerative calcified change. (2) A small tear might have occurred in the calcified portion, and the accumulated stress on the annulus allowed the small area of detachment. (3) There might have been an “unidentified cured infective process” in the remnant valve tissue. Because we could not find any fibrous tissue growth in the sewing cuff, we thought possible cause of PVL is weakening of the suture site by lack of fibrous tissue between the sewing cuff and annulus tissue.

If the PVL is small and the surrounding tissue is clear, direct suture closure or device closure maybe possible. If the leak is large or in multiple sites, the effective treatment is valve replacement.

## Conclusion

Careful follow-up is necessary for PVL in patients who have undergone valve replacement, even if many years ago. We successfully treated a rare case of multiple PVL that was detected 17 years after the second valve replacement.

## Consent

Written informed consent was obtained from the patient for publication of this case report and accompanying images. A copy of the written consent is available for review by the Editor-in-Chief of this journal.

## Competing interests

The authors declare that they have no competing interests.

## Authors’ contributions

TJ wrote the draft of the manuscript and obtained the written consent. DL performed the literature review and participated in the manuscript writing and helped to the final writing of the paper and gave final approval of the manuscript. All authors have read and approved the final manuscript.
